# Protection after stroke: cellular effectors of neurovascular unit integrity

**DOI:** 10.3389/fncel.2014.00231

**Published:** 2014-08-14

**Authors:** Rafael Andres Posada-Duque, George E. Barreto, Gloria Patricia Cardona-Gomez

**Affiliations:** ^1^Cellular and Molecular Neurobiology Area, Group of Neuroscience of Antioquia, Faculty of Medicine, Sede de Investigación Universitaria (SIU), University of Antioquia UdeAMedellín, Colombia; ^2^Departamento de Nutrición y Bioquímica, Facultad de Ciencias, Pontificia Universidad JaverianaBogotá D.C., Colombia

**Keywords:** stroke, NVU, BBB, CDK5, Rho GTPases, p120 catenin

## Abstract

Neurological disorders are prevalent worldwide. Cerebrovascular diseases (CVDs), which account for 55% of all neurological diseases, are the leading cause of permanent disability, cognitive and motor disorders and dementia. Stroke affects the function and structure of blood-brain barrier, the loss of cerebral blood flow regulation, oxidative stress, inflammation and the loss of neural connections. Currently, no gold standard treatments are available outside the acute therapeutic window to improve outcome in stroke patients. Some promising candidate targets have been identified for the improvement of long-term recovery after stroke, such as Rho GTPases, cell adhesion proteins, kinases, and phosphatases. Previous studies by our lab indicated that Rho GTPases (Rac and RhoA) are involved in both tissue damage and survival, as these proteins are essential for the morphology and movement of neurons, astrocytes and endothelial cells, thus playing a critical role in the balance between cell survival and death. Treatment with a pharmacological inhibitor of RhoA/ROCK blocks the activation of the neurodegeneration cascade. In addition, Rac and synaptic adhesion proteins (p120 catenin and N-catenin) play critical roles in protection against cerebral infarction and in recovery by supporting the neurovascular unit and cytoskeletal remodeling activity to maintain the integrity of the brain parenchyma. Interestingly, neuroprotective agents, such as atorvastatin, and CDK5 silencing after cerebral ischemia and in a glutamate-induced excitotoxicity model may act on the same cellular effectors to recover neurovascular unit integrity. Therefore, future efforts must focus on individually targeting the structural and functional roles of each effector of neurovascular unit and the interactions in neural and non-neural cells in the post-ischemic brain and address how to promote the recovery or prevent the loss of homeostasis in the short, medium and long term.

## Introduction

Neurological disorders are highly prevalent around the globe. In 2008, neurodegenerative disorders were responsible for 1% of disabilities worldwide (W.H.O., 2003). Strokes account for 55% of all neurological diseases and are considered the leading cause of permanent physical and mental disability (OMS, 2013). The primary risk factors of stroke include hyperlipidemia, hypertension, diabetes mellitus, and harmful habits, such as smoking and excessive alcohol consumption. The high incidence of strokes is related to an increased number of dementia cases and other emotional and cognitive disorders, such as depression and memory loss (Ovbiagele and Nguyen-Huynh, 2011). Death, physical deterioration, and altered quality of life are consequences of the natural history of strokes among patients who survive an ischemic event (Sacco, 1997, 1998; Feigin et al., 2003; Silva et al., 2006). Interestingly, the coexistence of cerebral ischemia and neurodegenerative pathologies profoundly impacts the development of dementia, suggesting a reciprocal interaction between ischemia and neurodegeneration (Nagy et al., 1997; Snowdon et al., 1997). These observations, along with the results of epidemiological studies that have indicated that Alzheimer’s disease (AD) and cerebrovascular diseases share similar risk factors (Breteler, 2000), have shifted interest to vascular factors as fundamental contributors to the pathogenesis of neurodegenerative diseases (de la Torre and Mussivand, 1993; Kalaria, 2000; Iadecola and Gorelick, 2003). This hypothesis has been supported by the experimental findings that showed that amyloid-beta (Aβ) peptide, which is commonly detected in AD patients, exhibits strong cerebrovascular effects and that ischemia-induced responses to hypoxia are potent modulators of cerebral amyloidogenesis (Iadecola, 2004). Both Aβ peptide and vascular risk factors deteriorate the structure and function of the neurovascular unit (NVU, consisting of the endothelium, glia, neurons, pericytes, and the basal lamina) (Mirra and Gearing, 1997; Snowdon et al., 1997; Breteler, 2000; Kalaria, 2000; Iadecola and Gorelick, 2003; Iadecola, 2004).

**Figure 1:**
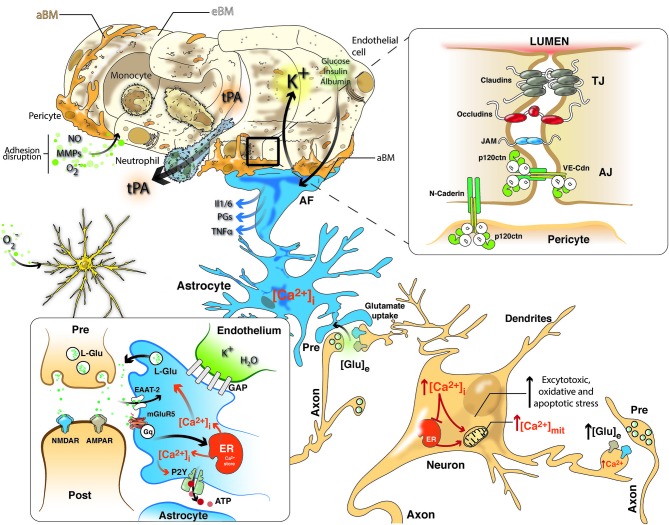
**NVU in cerebral ischemia includes a closely coupled signaling network of ECs, pericytes, astrocytes and neurons**. Neurovascular uncoupling consists of the expression of various cytokines and adhesion molecules on ECs, promoting leukocyte adherence and accumulation, which thereby initiates an inflammatory response. Further, breakdown of the BBB via NO, MMPs and ROS permits neutrophil extravasation into the ischemic tissue in response to chemokines produced by astrocytes, macrophages, and microglia. Glutamate-mediated excitotoxicity via NMDA and AMPA receptors induces the hyperstimulation of neurons and subsequent calcium influx, indicating several intracellular calcium stores (mitochondria and ER), which triggers dendritotoxic and cell death pathways. Intrinsic neurovascular responses after stroke are triggered by astrocytes via the spread of calcium waves, promoting homeostatic gliotransmission. In addition, glutamate uptake by astrocytes, which requires EAATs, represents the primary neuroprotective response after excitotoxicity. Alternatively, K^+^ and glucose uptake and insulin and albumin transport signal the modulation of neurovascular homeostasis. Tight junctions (TJs) consisting of claudins, occludins and JAM and adherens junctions (AJ) including members of the cadherin-catenin system maintain the integrity of the endothelium. Cell–cell adhesion may transmit intracellular signals that regulate paracellular permeability, contact-induced inhibition of cell growth and new vessel formation. After stroke, pericytes display deformed junctions and increased formation of pinocytic vesicles, which affect the polarization of astrocyte end-feet (AF), detected as the defective deposition of the astrocyte-derived basement membrane (aBM). Deposition of the endothelium-derived basement membrane (eBM) is affected by the lack of pericytes. Moreover, modifications of the molecular organization and intracellular signaling of junction proteins may exert complex effects on vascular homeostasis.

The NVU acts as a guardian of cerebral homeostasis. Neurons, glia, the perivascular space, and the endothelium are closely interrelated to maintain the homeostasis of the brain microenvironment (Iadecola, 2010), regulate blood flow, modulate the exchange across the blood-brain barrier (BBB), contribute to immune vigilance and provide trophic support to the brain (Iadecola, 2010). Substantial evidence has shown that cerebrovascular dysfunction is implicated in not only cognitive impairment (such as that of cognitive origin) but also neurodegenerative diseases, such as AD (Chui et al., 1992; Alavi et al., 1998; Kalaria, 2000; Iadecola, 2004; Simpkins et al., 2005; Hachinski et al., 2006; Pendlebury et al., 2012). Ischemic stroke is exacerbated by several risk factors that affect the function and structure of blood vessels in the brain and cells associated with the NVU, reducing the ability of the brain parenchyma to repair due to the rupture of the BBB, the loss of brain blood flow regulation, oxidative stress, inflammation, and the loss of neuronal connections, ultimately increasing brain dysfunction (Deane et al., 2003; Ohab et al., 2006; Konsman et al., 2007; Weber et al., 2007; Bell et al., 2009; Wolburg et al., 2009). The study of stroke has focused on understanding the molecular and pathophysiological mechanisms of neuronal death, recovery and pharmacological intervention strategies, as well as clinical and epidemiological characteristics (Silva et al., 2006). In addition, several molecular targets associated with endothelial dysfunction and cardio-cerebrovascular risk, including CDK5, Rho GTPases, and cell adhesion proteins, are described below and presented in a hypothetical schematic in Figure 1 to explain and propose a potential neuroprotective approach for stroke.

## Cerebral ischemia

Cerebral ischemia is a type of stroke characterized by a transient or permanent decrease in blood flow as a result of the thrombotic or embolic occlusion of one or more cerebral arteries. Depending on the ischemic characteristics (i.e., the duration of reduced blood flow and the infarction site), this disease can result in several clinical manifestations, including paralysis or hemiplegia, aphasia, and memory and learning impairment, among others (Kemp and Mckernan, 2002).

In focal cerebral ischemia, a hypoperfusion gradient is generated, leading to the activation of diverse cell types involved in survival and cell death mechanisms (Lipton, 1999; Barreto et al., 2011, 2012). Within minutes after ischemia, the activated microglia induces cytokine and adhesion molecules; hours to days, the endothelium responds by increasing angiogenesis; in days, weeks to months, astrocytes expressing GFAP (glial-fibrillary acidic protein) generate glial scar; and the neurons trigger axonal sprouting, dendrite outgrowth, spine morphogenesis (Iadecola, 1997; Rami et al., 2008; Barreto et al., 2012). The region suffering the most severe degree of hypoperfusion, referred to as the ischemic core, progresses rapidly toward irreversible damage via necrosis. The remaining hypoperfused tissue displays altered mechanisms of blood flow autoregulation and is known as the penumbra (Baron, 2001; Moustafa and Baron, 2008). In this peri-infarct zone, neurons display functional alterations and minimal metabolic activity to preserve their structure, but these neurons ultimately advance toward apoptotic death (Mies et al., 1993; Moskowitz et al., 2010). Accordingly, the penumbra is not only a functionally affected tissue but is also potentially recoverable and, as such, represents a key target for therapeutic intervention for cerebral ischemia (Baron et al., 1995). However, unless perfusion is restored or the cells surrounding the injury site become relatively resistant, the cells in the penumbra die via apoptosis within a few hours (Lo, 2008b).

An interruption in the blood supply to the brain during ischemia results in oxygen-glucose deprivation and, consequently, reduced energy available for brain cell functions (Dingledine et al., 1999). In particular, neurons become incapable of maintaining the transmembrane ion gradients necessary for their function and homeostasis (Szydlowska and Tymianski, 2010). This event leads to excessive neuronal depolarization, an increase in the release of excitatory neurotransmitters and pro-inflammatory molecules, a reduction in the reuptake of these neurotransmitters from the extracellular space in penumbra and a GABAergic and dopaminergic misbalance in exo-focal areas (Sabogal et al., 2014). Altogether, these pathological mechanisms induce an excessive intracellular accumulation of ions such as Na^+^ and Ca^2+^ and, simultaneously, the deregulation of multiple signaling pathways, activating catabolic processes mediated by proteases, lipases, and nucleases, which interrupt neuronal function and induce cell death (Dingledine et al., 1999; Szydlowska and Tymianski, 2010; Barreto et al., 2011). Increased extracellular glutamate concentrations in central nervous system (CNS) pathologies (including stroke, epilepsy and certain neurodegenerative diseases) result in the local hyperactivation of ionotropic glutamate receptors, thereby triggering neuronal cell death via excessive Na^+^ and Ca^2+^ influx into neurons (Olney, 1969; Choi, 1987; Sattler and Tymianski, 2001; Greenwood and Connolly, 2007). This event is known as glutamate-mediated excitotoxicity (Lipton, 1999; Mehta et al., 2007). In the core, glutamate excitotoxicity rapidly evolves to necrosis due to ATP depletion and increased Na^+^ and water influx, whilst in the penumbra, where the damage seems to be less severe, glutamate excitotoxicity produces neuronal apoptosis (Bonfoco et al., 1995; Kelly et al., 2003; Lo, 2008a; Rami et al., 2008). Glutamate-induced dendritotoxicity results in microtubule disruption and the calcium-dependent loss of MAP2 and contributes to dendritic dysfunction in acute hippocampal slices (Bindokas and Miller, 1995; Hoskison and Shuttleworth, 2006; Hoskison et al., 2007) and nearly complete dendritic loss in cortical neuron cultures (Bosel et al., 2005; Ma et al., 2009). Toxic cytoplasmic calcium concentrations during ischemia can occur due to the release of calcium from internal stores via physical damage to the mitochondria and endoplasmic reticulum (ER) or the malfunction of receptors and channels present in their membranes (Loew et al., 1994; Paschen and Doutheil, 1999). The accumulation of intramitochondrial Ca^2+^ reduces ATP synthesis, and increased ATP usage has been suggested to be a primary cause of cell death (Schinder et al., 1996). The dysregulation of Ca^2+^-ER homeostasis following ischemia involves two phases: the accumulation of Ca^2+^ in ER stores and the subsequent release of Ca^2+^ from the ER following ischemia/reoxygenation (Chen et al., 2008). It has been suggested that Ca^2+^ released from the ER via IP3R can enter the adjacent mitochondria and trigger cytochrome c release (Rizzuto and Pozzan, 2006). The increase in the cytoplasmic calcium concentration triggers neurotoxic cascades, including the uncoupling of mitochondrial electron transfer from ATP synthesis and the activation and hyperstimulation of enzymes, such as calpains and other proteases, protein kinases, neuronal nitric oxide synthase (nNOS), calcineurin and endonucleases (Szydlowska and Tymianski, 2010).

In recent studies, we found that glutamate-mediated excitotoxicity decreases the number and length of dendrites and induces tau hyperphosphorylation (Posada-Duque et al., 2013). The hyperphosphorylation of tau, a neurodegeneration marker, is also increased by global and transient focal cerebral ischemia, causing alterations in spatial memory (Castro-Alvarez et al., 2011; Céspedes et al., 2013). At the cellular level, hyperactivated GSK3 protein is associated with alterations in microtubule assembly, and the activity of RhoA has been associated with the retraction of the actin cytoskeleton (Céspedes-Rubio et al., 2010). Damage to the neuronal cytoskeleton can be considered the primary cause of the loss of protein transport and neuronal stability in cerebral ischemia. Microtubule disassembly occurs after ischemia and plays an important role in the pathophysiology of this disease (Pettigrew et al., 1996). Alterations in the cytoskeleton reflect, in part, processes of protein degradation and aggregation following ischemia (Kühn et al., 2005). For example, we found that the loss of Rac activity participates in the progression of cerebral ischemia-induced neurodegeneration and is closely associated with cognitive disorders (Gutiérrez-Vargas et al., 2010). In addition, MAP2 degradation and tau aggregation and hyperphosphorylation are considered biomarkers of ischemic damage (Pettigrew et al., 1996; Gutiérrez-Vargas et al., 2010).

## NVU: the guardian of cerebral homeostasis

The BBB is a highly specialized endothelial brain structure composed of a specialized neurovascular system. Through pericytes, astrocytes, and microglia, the BBB separates components in circulating blood from neurons. Moreover, the BBB maintains the chemical composition of sodium, anion carbonate, glucose, lactate, essential amino acids, insulin, enkephalins, and arginine-vasopressin, which is required for the proper function of neuronal circuits in the adult brain (Zlokovic, 2008). The endothelium, the site of the anatomical BBB, neurons, and non-neuronal cells (e.g., pericytes, astrocytes, and microglia) together with the basal lamina form a functional unit, often referred to as the NVU, which participates in the establishment and function of the BBB (Lo et al., 2003; Hawkins and Davis, 2005). Cerebral endothelial cells (ECs) significantly differ from non-cerebral ECs in the following aspects: (i) the presence of intercellular tight junctions (TJs); (ii) low levels of transcytosis (pinocytosis) and paracellular diffusion of hydrophilic compounds; (iii) a high number of mitochondria, associated with increased metabolic activity; and (iv) the polarized expression of membrane receptors and transporters, which are responsible for the active passage of nutrients (Brightman and Kadota, 1992; Petty and Lo, 2002). Consistently, the permeability of plasma and ionic compounds in the cerebral endothelium is highly restricted and regulated—a phenomenon evidenced by a high trans-endothelial electric resistance (TEER; Petty and Lo, 2002; Abbott et al., 2006). Another component of the BBB, the basal lamina, is classified according to the cell source. The basal lamina of the endothelium is composed of three layers: one contains laminin-4 and -5 derived from ECs, one contains laminin-1 and -2 derived from astrocytes, and the other contains collagen IV derived from both cell types (Perlmutter and Chui, 1990).

The role of astrocytes in BBB function and integrity has been well documented; however, their molecular mechanisms of action remain unclear (Anderson et al., 2003; Giffard and Swanson, 2005; Thoren et al., 2005; Barreto et al., 2011). Astrocytes form structures called “astrocyte end-feet” that communicate with neurons, pericytes, and ECs. This cell population is very important because it modulates synaptic transmission and processes of neuronal plasticity (Ullian et al., 2001; Allen and Barres, 2005; Kucukdereli et al., 2011). These cells also maintain homeostasis by releasing sodium after action potentials, maintaining metabolic homeostasis via insulin, glucose and lactate signaling, and releasing hormones in response to various behaviors (Nedergaard et al., 2003; Newman, 2003; Abbott et al., 2006). It has been suggested that astrocytes contribute support to the BBB via the release of factors including glial cell line-derived neurotrophic factor (GDNF), angiopoietin-1, and angiotensin II (Lee et al., 2003; Hori et al., 2004; Wosik et al., 2007).

Complementarily, pericytes and the basal lamina surround ECs along the cerebral microvasculature. This cerebral microvasculature is enriched in pericytes, and the ratio of pericytes to ECs correlates to the barrier capacity of the endothelium. In addition, pericytes are actively involved in the maintenance of vessel integrity, vasoregulation, and the regulation of BBB permeability (Lindahl et al., 1997; Peppiatt et al., 2006; Armulik et al., 2010). Finally, it has been observed that neuronal projections are closely related with the cerebral endothelium (Hamel, 2006; Drake and Iadecola, 2007; Weiss et al., 2009). These perivascular neurons use perivascular astrocytes and pericytes to modulate brain blood flow and vessel dynamics (Hamel, 2006; Wang and Bordey, 2008; Sofroniew and Vinters, 2010). Recently, it has been demonstrated that the close proximity of these different cell types to neurons facilitates effective paracrine regulation, which is critical for the normal function of the CNS; however, the impaired paracrine regulation induces the development of neurological diseases (Berzin et al., 2000; Zlokovic, 2005). The normal function of the CNS requires the regulation of neurovascular coupling, microvascular hemodynamics and permeability, matrix interactions, neurotransmitter inactivation, neurotrophic support, and processes of neurogenesis and angiogenesis (Boillée et al., 2006; Deane and Zlokovic, 2007). It is currently accepted that the BBB limits the entrance of blood components into the cerebral parenchyma. Therefore, injury to any part of the neurovascular unit could permit the extravasations of vascular inflammatory cells and proteins to brain tissue (del Zoppo, 2006). Extravasation of the BBB due to cerebral ischemia, trauma, neurodegenerative processes, or vascular disorders allows these neurotoxic products to compromise synaptic and neuronal function (Hawkins and Davis, 2005; Zlokovic, 2005; Abbott et al., 2006). When the brain blood flow is interrupted, brain functions halt within seconds, and neuronal damage occurs after a few minutes (Girouard and Iadecola, 2006). An appropriate vasculature-neuron ratio is critical for normal brain function. It has been estimated that nearly every neuron in the brain has its own capillary (Zlokovic, 2005). The total length of the capillaries in the human brain is approximately 400 miles, and the surface area available for molecular transport is approximately 20 m^2^ (Begley and Brightman, 2003). The length of the brain capillaries is reduced in neurodegenerative disorders, such as AD and cerebral ischemia (Bailey et al., 2004; Wu et al., 2005). These vascular reductions diminish the transport of energy substrates and nutrients across the BBB and reduce the clearance of potentially neurotoxic substances during brain injury.

## BBB dysfunction in cerebral ischemia

In a variety of neurological diseases (e.g., AD, ischemia, Huntington’s disease, Parkinson’s disease, and ALS), BBB dysfunction has been detected not only as a late event but also at early stages of disease progression. There are two types of BBB dysfunction involving increased permeability: passive diffusion with edema formation and massive cellular infiltration across the BBB (Weiss et al., 2009). Acute obstruction of brain blood vessels due to coagulation during cerebral ischemia involves a complex series of cellular and molecular events in the brain parenchyma (del Zoppo and Mabuchi, 2003). The sequence of events from ischemia to reperfusion begins with perivascular inflammation and increased BBB permeability, which greatly contribute to brain damage, thus supporting the notion that cerebral ischemia is primarily a vascular disorder (Benchenane et al., 2004). The release of oxidants, proteolytic enzymes, and proinflammatory cytokines alters BBB permeability, leading to brain edema (Dirnagl et al., 1999). In addition, metalloproteinases (MMPs) released by activated leukocytes affect NVU integrity, degrading the basal lamina and the proteins involved in cell-cell adhesion (Hamann et al., 1995; Rosenberg et al., 1998; Asahi et al., 2001). MMP-9 is particularly critical during this process, as previous studies have demonstrated that BBB integrity is maintained after cerebral ischemia in MMP-9-deficient mice (Asahi et al., 2001; Montaner et al., 2001). However, increased trans-endothelial migration of leukocytes is characteristic during cerebral ischemia (Lindsberg et al., 1996; Planas et al., 2006). Following ischemia, circulating leukocytes release inflammatory cytokines and activate various cell types within the NVU. For example, the increased expression of intercellular adhesion molecule-1 (ICAM-1) by ECs facilitates the trans-endothelial migration of leukocytes (Lindsberg et al., 1996). Furthermore, it has been demonstrated that treatment with anti-ICAM-1 antibodies reduces infarct size in rat models of cerebral ischemia (Zhang et al., 1995). Another process of BBB dysfunction that increases damage during cerebral ischemia is the secretion of TGFβ by astrocytes, which diminishes endothelial capillarity, fibrinolytic enzyme expression, and basal lamina proteolysis. Additionally, BBB disruption coincides with the induction of aquaporin 4 (AQP4) expression and the presence of reactive astrocytes in the perivascular glial cells (Tran et al., 1999; Lo et al., 2003; Tomás-Camardiel et al., 2005; Vakili et al., 2005). Several chemical agents circulating in the plasma or secreted from cells associated with the BBB are capable of increasing brain endothelial permeability and impairing its transport and metabolic functions (Laflamme et al., 1999; Abbott, 2000; Webb and Muir, 2000). These agents include histamine, serotonin, glutamate, purine nucleotides (ATP, ADP, and AMP), adenosine, platelet-activating factor, phospholipase A2, arachidonic acid, prostaglandins, leukotrienes, tumor necrosis factor-α (TNFα), free radicals and nitric oxide (Laflamme et al., 1999; Abbott, 2000; Webb and Muir, 2000; Tan et al., 2002; Stolp and Dziegielewska, 2009). Bradykinin, which is produced during inflammation in stroke, acts on endothelial and astroglial bradykinin B2 receptors, leading to an increase in intracellular Ca^2+^ concentrations. In astrocytes, this event can trigger the production of interleukin (IL)-6 and IL-1 via nuclear factor-κB (NF-κB) activation (Deli et al., 1995; Schwaninger et al., 1999; Didier et al., 2003; Perry et al., 2003). The role of the microglia in pathological NVU activity has been proposed to induce damage to the endothelium and the basal lamina via receptors for nucleotides, such as ATP, and opening new routes of communication between ECs, pericytes, astrocytes and microglia is important for BBB repair (Deitmer et al., 2001; Andersson et al., 2005). However, recent studies have demonstrated that enhancing the activation of microglia promotes tissue repair and remodeling after stroke by decreasing the levels of the inflammatory markers IL-1β and TNF-α and increasing IL-1ra, IL-10, and arginase 1 expression (Gelosa et al., 2014; Shin et al., 2014).

Apparently, the NVU performs a coordinated response after an ischemic insult to maintain and re-establish blood flow over time, thus reducing damage to tissue and neurons. Aside from the notion that occlusion affects vulnerable regions, this hypothesis supports the need to understand the interactions between each component of the NVU in the pathophysiology of cerebral ischemia and recovery following therapy (Nedergaard et al., 2003; Zonta et al., 2003; Iadecola, 2004; Abbott et al., 2006; Koehler et al., 2009). The strategy of protection of neuronal function in humans was not successful in preventing damage progression, indicating that there is a mechanism that is inherent to NVU coupling. Moreover, there is a relapse of the NVU function that leads to vascular dementia (del Zoppo, 2010), despite the intrinsic recovery of the tissue and of cognitive abilities. Although the use of neuroprotective agents has generally failed as a potential therapeutic strategy following cerebral ischemia in translational trials, thrombolysis using tissue plasminogen activator (tPA) continues to be the gold standard intervention during the acute phase after stroke (van der Worp and van Gijn, 2007). Importantly, neuroprotective agents should be administered early by the emergency medical system (EMS) to improve neuroprotection in the long term (Saver, 2013), which also helps to prevent the impairment of the integrity of the NVU and avoid cerebral dysfunction. Therefore, in an attempt to understand the interdependency of the components of the NVU, its integrity and how to protect it, we will focus on certain implicated tissue and cellular effectors.

## Intercellular junctions in the BBB

ECs are primarily connected via junction complexes, which consist of TJs and adherens junctions (AJ; Hawkins and Davis, 2005). Although gap junctions (GJ) have also been found in the BBB, their role remains unclear (Nagasawa et al., 2006). TJs are primarily the junctions that confer a low paracellular permeability and TEER to the BBB (Bazzoni et al., 2005). TJ physiology is complex. TJ proteins and their adaptor molecules, which are connected to the cytoskeleton, are often affected during acute and chronic brain disease (Wolburg and Lippoldt, 2002). Occludin was the first integral protein to be identified in TJs of ECs involved in the BBB. It has been demonstrated that the N-terminal domain of occludin plays an important role in TJ assembly and the maintenance of barrier function (Bamforth et al., 1999). In contrast, deletion of the occludin gene in mice results in a complex phenotype, including a delay in postnatal growth, due to the efficient permeability of the barrier caused by a diminished TEER and an increased paracellular flow of macromolecules (Bamforth et al., 1999; Saitou et al., 2000). Occludin is also vulnerable to degradation by MMPs (Rosenberg and Yang, 2007). Damage caused by reperfusion in ischemic models in rodents leads to a biphasic opening of the BBB—a process directed by MMP-2 activation during the acute phase that occurs for several hours after reperfusion (Zlokovic, 2006). This initial transient opening is followed by more intense damage to blood vessels from 24 to 48 h after reperfusion, which is associated with the expression of MMP-9 and MMP-3 (Suzuki et al., 2007). MMPs can also degrade basal lamina components, such as fibronectin, contributing to BBB rupture (Cheng et al., 2006; Zlokovic, 2006). Claudins are a family consisting of more than 20 members that generate TJ chains via hemophilic claudin-claudin interactions. Claudin −5, −3, and −12 are located on the BBB, whereas the localization of claudin-1 is controversial. With regard to the function of claudins at the BBB, it has been found that each claudin isoform regulates the diffusion of a group of molecules of a particular size (Wolburg and Lippoldt, 2002; Lee et al., 2003; Nitta et al., 2003). Although claudin-5 protein expression is low in brain ECs, during BBB rupture, this protein is a target of MMP-2- and MMP-9-mediated degradation after ischemia and, together with occludin, is found near astrocytes (Yang et al., 2006). Integral TJ membrane proteins are attached to the cytoskeleton by cytoplasmic multi-domain scaffolding proteins, such as ZO-1, ZO-2, and ZO-3 (Hawkins and Davis, 2005; Hawkins et al., 2007), and these proteins are also targets of MMP-mediated degradation during CNS injury. Essentially, BBB opening during cerebral ischemia is clearly induced by the MMP-mediated degradation of TJs; however, it has recently been demonstrated that MMPs also participate in the regulation of neurogenesis and angiogenesis during the recovery phase after injury (Fujioka et al., 2012). This beneficial characteristic of MMP activity was demonstrated in a late event ischemia model (7–14 days), in which treatment with MMP inhibitors 7 days after stroke suppressed neurovascular remodeling, increased injury, and impaired cognitive function. It has been suggested that these effects are primarily due to a decrease in VEGF availability. Therefore, clarification regarding the therapeutic potential of MMP inhibition for different brain pathologies has been recommended (Zhao et al., 2006). Alternatively, AJs typically crosstalk with TJs. AJs are a type of cell-cell adhesive contact found in many tissues, and AJs use a cadherin dimer as the primary mediator of cell-cell adhesion. Endothelial permeability is regulated in part by the dynamic opening and closing of AJs (Bazzoni and Dejana, 2004). In ECs, AJs primarily consist of vascular endothelial cadherin (VE-cadherin), an endothelium-specific member of the cadherin that binds to p120 catenin, β-catenin, and plakoglobin via its intracytoplasmic domain (Dejana et al., 2008). Multiple endogenous pathways that increase vascular permeability affect the function and organization of VE-cadherin (Bazzoni and Dejana, 2004). Loss of VE-cadherin is accompanied by an increase in vascular permeability and leukocyte diapedesis, and internalization and cleavage of VE-cadherin can disassemble AJs at cell-cell junctions (Lampugnani and Dejana, 2007). Changes in AJ proteins expression contribute to increased BBB permeability and leukocyte infiltration into the CNS (Allport et al., 1997; Johnson-Léger et al., 2000). Several mechanisms by which VE-cadherin regulates endothelial function have been proposed, such as the direct activation of signaling molecules to maintain survival and the organization of the actin cytoskeleton (e.g., PI3 kinase and Rac), the regulation of gene transcription co-factors (e.g., p120 catenin, β-catenin), the formation of complexes with growth factor receptors, including the vascular endothelium growth factor receptor-2 (VEGFR-2), and the modulation of VEGFR-2 signaling (Bazzoni and Dejana, 2004). The roles of AJs in the functional and morphological changes in the BBB during cerebral ischemia have not been addressed in depth. The direct activation of oxygen-sensitive transcription factors, such as HIF-1 and reactive oxygen species (ROS), can directly activate transcription factors, such as NF-κB, which alter AJ formation. The increase in intracellular calcium levels that characterizes cerebral ischemia can lead to the transduction of signals that regulate AJ and TJ transcription, consequently inducing changes in BBB function (Brown and Davis, 2002). Based on these studies, it is necessary to develop strategies to maintain NVU structure and function after cerebral ischemia by focusing on obtaining a deeper understanding of the cell-cell junctions present in the BBB.

## Rho GTPases in ECs regulate BBB integrity

The importance of the cytoskeleton in BBB integrity and establishment was initially demonstrated in mice deficient in the actin-binding protein dystrophin (Nico et al., 2003). These mice displayed increased vascular permeability, as the ECs and astrocytes formed a disorganized actin cytoskeleton, as well as altered subcellular localization of junction proteins in the endothelium and aquaporin-4 at astrocyte end-feet (Nico et al., 2003). These findings demonstrated that the arrangement of actin filaments and their junctions to TJs and/or AJs are critical for normal BBB function. The actin cytoskeleton plays an essential role in maintaining the barrier function of the endothelium because it determines cell shape, facilitates cell-matrix adhesion, and participates in the regulation of junction complexes (Dejana, 2004). It has been determined that the dynamics and structure of the actin filaments are primarily regulated by Rho GTPase proteins (Ridley, 2001; Lampugnani et al., 2002; Aspenstrom et al., 2007; Aghajanian et al., 2008; Vandenbroucke et al., 2008). The Rho family of GTPases belongs to the Ras superfamily, which consists of low molecular weight, monomeric G-proteins. Rho GTPases have recently been classified into six subfamilies: RhoA, Rac1, Cdc42, Rnd, RhoBTb, and Rho/Miro (Ridley, 2001; Lampugnani et al., 2002; Aspenstrom et al., 2007; Aghajanian et al., 2008; Vandenbroucke et al., 2008). Specifically, RhoA induces the formation of actin stress fibers and focal adhesions, Rac1 stimulates lamellipodial protrusions (membrane ruffling), and Cdc42 promotes the formation of filopodia (actin microfilaments) (Ridley and Hall, 1992; Nobes and Hall, 1995). In *in vivo* experiments, multiple vasoactive agents improved endothelial barrier integrity by forming a dense F-actin band via changes in the activities of various Rho GTPases (Garcia et al., 2001; Temmesfeld-Wollbrück et al., 2007; Tauseef et al., 2008). Initially, RhoA activation was found to be important because RhoA regulates cell contraction and endothelial hyperpermeability (Sun et al., 2006). In subsequent years, attention was directed toward the involvement of Rac and Cdc42 in the assembly and stability of inter-endothelial junctions. Furthermore, a recently discovered role of RhoA and its downstream effector ROCK in barrier maintenance was identified (Broman et al., 2007; Fu and Birukov, 2009). An important role of Rho GTPases in BBB regulation is that they act as mediators of barrier damage or protection (Beckers et al., 2010). Although Rac1 primarily modulates the stability of cell-cell junctions, its activity on NADPH oxidase-mediated ROS generation participates in BBB damage after injury (Broman et al., 2007; Monaghan-Benson and Burridge, 2009). Likewise, RhoA plays a dual role in barrier regulation. For example, RhoA exerts a protective function under basal conditions, but it is involved in BBB dysfunction after EC activation by thrombin (Broman et al., 2007; van Nieuw Amerongen et al., 2008). In particular, RhoA activity is involved in the progression of neuronal death during cerebral ischemia (Semenova et al., 2007). In our study, we found that there is a differential regulation of Rho GTPases due to global and transient focal cerebral ischemia. We demonstrated that Rho/ROCK inhibition is involved in neuroprotection (Castro-Alvarez et al., 2011). Additionally, we found that treatment with statins—a class of drugs used to reduce cholesterol—diminished RhoA activity, which is associated with decreased levels of neurodegeneration markers and neuronal death (Céspedes-Rubio et al., 2010). Some of the mechanisms associated with the neuroprotective role of statins include the downregulation of RhoA activity and the subsequent induction of endothelial nitric oxide synthase (eNOS) activity (Wang et al., 2008). Finally, we discovered that Rac1 activity is involved in long-term recovery from neurodegeneration after cerebral ischemia (Gutiérrez-Vargas et al., 2010). Because the function of Rho GTPase is essential for NVU regulation (including the endothelium, neurons, and adhesion proteins), the challenge of maintaining BBB integrity lies in determining the complex regulation of Rho GTPases, which will provide indications for new therapeutic strategies for ischemic injury.

## CDK5, a target for cerebral ischemia

CDK5 is a serine-threonine kinase that participates in neuronal development and function and is involved in the regulation of various processes, such as neuritogenesis, synapse formation, and synaptic transmission (López-Tobón et al., 2011; Su and Tsai, 2011). CDK5 also plays an important role in the regulation of apoptosis during development, which is essential for the pruning and fine-tuning of neural connections, and is involved in cognitive functions, such as memory and learning. In addition, the deregulation of CDK5 activity is associated with neuronal death in neurodegenerative diseases (Dhavan and Tsai, 2001; Lai and Ip, 2009). CDK5 activity is primarily regulated by p35 and p39, and the phosphorylation of CDK5 at Ser159 and Tyr15 increases its activity (Zukerberg et al., 2000; Grant et al., 2001). Cleavage of p35 by calpains results in the formation of a CDK5/p25 complex, leading to considerable sustained abnormal CDK5 kinase activity (Cheung et al., 2006). Morphological characteristics, such as the density and morphology of dendritic filaments, and biochemical characteristics, such as the composition of postsynaptic density scaffold proteins and the number of neurotransmitter receptors, determines the properties of synaptic activity. Many synaptic proteins isolated from adult brain synaptosomes are putative CDK5 substrates, rendering CDK5 a synapse regulator (Collins et al., 2005). CDK5 is involved in cognitive deterioration in cerebral ischemia and neurodegeneration (Slevin and Krupinski, 2009; Menn et al., 2010). In addition to the many studies showing that CDK5 hyperactivation is involved in tau hyperphosphorylation and the subsequent development of dementia in neurodegenerative diseases (Ko et al., 2001; Camins et al., 2006), multiple pieces of evidence have implicated CDK5 in the progression of cerebral ischemia pathology. Increased expression levels of CDK5 and its activator p35 are detected in peri-infarct neurons following middle cerebral artery occlusion (MCAO; Green et al., 1997; Mitsios et al., 2007). In cerebral ischemia models, pharmacological inhibition of CDK5 significantly reduces the infarct size after 24 h of reperfusion (Weishaupt et al., 2003). AD and other tauopathies, such as cerebral ischemia, are characterized by the hyperphosphorylation of the protein tau (Grundke-Iqbal et al., 1986; Avila et al., 2004). Particularly, CDK5, along with its respective activators, phosphorylates tau at epitopes that are associated with neurodegenerative processes. Deregulated CDK5 activity is produced by the membrane-released CDK5/p25 complex, leading p25 to induce an atypical and sustained activation of CDK5, which leads to the hyperphosphorylation of its substrates that are associated with disease progression. Overexpression of p25 in neuronal cultures produces cytoskeletal damage, tau hyperphosphorylation, and apoptosis (Patrick et al., 1999; Wen et al., 2008). Because CDK5 inhibition protects against neuronal death and reduces tau hyperphosphorylation (Lopes et al., 2007; Piedrahita et al., 2010) and because the atypical activation of CDK5 is a fundamental component of the progression of the neurofibrillary pathology, CDK5 inhibition has been proposed as a potential therapeutic strategy for neurological diseases. During glutamate-mediated excitotoxicity, the excessive influx of Ca^2+^ can activate the Ca^2+^-dependent protease calpain. Calpain is involved in the degradation of numerous enzymes and cytoskeletal components, thus linking its activity to a variety of intracellular events involved in CNS alterations associated with excitotoxicity, such as hypoxia, ischemia, epilepsy, and AD (Ray and Banik, 2003). Recently, it has been shown that CDK5 phosphorylates the NMDA receptor subunit NR2A, causing neuronal death in the CA1 area of the hippocampus in a transient global cerebral ischemia model. This finding suggests a possible therapeutic approach in which CDK5 inhibition targets glutamate receptors (Wang et al., 2003). Alternatively, previous studies have reported the involvement of CDK5 in the progression of neurovascular pathology. Importantly, patients suffering from cerebral ischemia display strong up-regulation of CDK5, p35, and p25 in peri-infarct blood vessels; additionally, human brain microvascular endothelial cells (HBMECs) subjected to glucose deprivation display hyperactivation of CDK5, suggesting a crucial role of CDK5 in the microvasculature in response to cerebral ischemia (Slevin and Krupinski, 2009).

## CDK5 in the endothelium and astrocytes

Early investigation of CDK5 function in ECs included studies that demonstrated decreased CDK5 expression when EC proliferation was inhibited by angiotensin. In a 2008 study, Liu and colleagues demonstrated that mitogenic growth factors activated the PI3K/Akt pathway, followed by CDK5-mediated phosphorylation of PIKE-A (a factor that hyperactivates phosphatidylinositol 3-kinase (PI3K)) in glioblastoma cells. These findings suggest that CDK5 may play a significant role in the regulation of EC proliferation (Sharma et al., 2004; Liu et al., 2008). One recently emergent function of CDK5 is that it acts as a regulator of the endothelium during angiogenesis and EC migration, thus contributing to various pathological conditions. Therefore, CDK5 has been proposed as a novel target for anti-angiogenic therapy (Liebl et al., 2010). In *in vivo* and *in vitro* models, such as neovascularization of the mouse cornea and endothelial tube formation, pharmacological inhibition of CDK5 resulted in reduced endothelial cell motility and angiogenesis, suggesting an effect independent of its previously characterized functions in neurons and a specific role of CDK5 signaling in the endothelium (Liebl et al., 2011). In contrast to its activity in neurons, the reduction in endothelial cell motility induced by CDK5 inhibition was not caused by altering the function of focal adhesions or microtubules, but rather by a reduction in lamellipodia formation (Liebl et al., 2010). CDK5 inhibition diminished Rac1 activity, leading to actin cytoskeleton disorganization, which suggests that CDK5 exerts its effects on endothelial cell migration via Rac1. Taken together, these findings indicate CDK5 as a pharmacologically accessible target for anti-angiogenic therapy and provide a basis for a new therapeutic strategy for cerebral ischemia.

One event that follows cerebral ischemia is the formation of new blood vessels. Recently, it has been shown that targeting p35/CDK5 using a specific peptide inhibitor (CIP) in a hypoxia model preserves cell motility and temporal control of actin cytoskeletal dynamics and protects and promotes angiogenesis (Bosutti et al., 2013). Although new vessels are the source of trophic factors during the early stages of brain parenchyma recovery, sustained angiogenesis becomes a physical barrier to neural circuit formation and connection refinement (Liebl et al., 2010). In addition, a previous study proposed roscovitine (an inhibitor of CDK5 and other CDKs) administration for the prevention of endothelial activation and the leukocyte-EC interaction during parenchymal leukocyte infiltration (Berberich et al., 2011). These studies could expand the use of CDK5 inhibition because of its promising anti-inflammatory effects, in addition to maintaining BBB integrity. Finally, CDK5 regulates eNOS enzyme activity. eNOS is an essential enzyme responsible for the production of endothelium-derived NO, which is a key molecule that performs multiple functions, including vascular homeostasis, angiogenesis, and cell cycle regulation (van Haperen et al., 2002; Desjardins and Balligand, 2006; Bonnin et al., 2012). CDK5 phosphorylates eNOS at Ser113 and Ser116, thus regulating nitric oxide (NO) levels (van Haperen et al., 2002; Desjardins and Balligand, 2006; Cho et al., 2010; Lee et al., 2010). eNOS deregulation contributes to the pathophysiology of multiple diseases, such as atherosclerosis, hypertension, and cancer (Desjardins and Balligand, 2006). It is well known that eNOS phosphorylation modulates its activity (Harris et al., 2001; Mount et al., 2007). Phosphorylation of eNOS by CDK5 is associated with decreased NO production (Cho et al., 2010). This mechanism may explain how NO is maintained at minimal levels at a basal state in ECs and how CDK5 contributes to the pathogenesis of diseases associated with reduced NO release from ECs. However, further studies are required to clarify these issues.

A few studies have shown that CDK5 and p35 are expressed in astrocytes in the adult brain. Additionally, it has been demonstrated that the activity of CDK5 and its activator p35 participate in the process of scratch-wound migration in a wound-healing model via the regulation of microtubules in primary astrocyte cultures (He et al., 2007). Likewise, increased CDK5 activity is involved in tau hyperphosphorylation, oxidative stress and the degeneration of astrocytes in a murine senescence model (García-Matas et al., 2008). Primarily, in GFAP/tau transgenic mice, a unique astrocytic tau pathology model, astrocytes have been identified as playing a role in the neurodegenerative pathology that is associated with CDK5 hyperphosphorylation (Forman et al., 2005). To date, there have been few findings involving CDK5 in astrocytic and endothelial function. Therefore, further studies of the possible role that this kinase might play in these cell types, as well as in other brain neurovascular cell types, namely microglia and oligodendrocytes, should be addressed.

## Regulation of cell adhesion by p120 catenin in NVUs

The neural component of the NVU is partially regulated by a group of cell adhesion molecules. Specifically, the catenin/cadherin system interacts with and regulates the architecture of the actin cytoskeleton (Salinas and Price, 2005). Neuronal morphology and plasticity require a dynamic actin cytoskeleton (Dillon and Goda, 2005). p120 catenin, in particular, regulates synapse development and morphology via Rho GTPases, which control actin cytoskeleton dynamics (Elia et al., 2006). Simultaneously, CDK5, via the phosphorylation of δ-catenin (a p120 catenin family member), participates in the processes of synaptic transmission (Poore et al., 2010). Therefore, with respect to neurons, it is possible to suggest an association between CDK5 activity and modulation of p120 catenin that facilitates neural plasticity processes. However, the possible relationship between the functions of CDK5 and p120 catenin has not been explored in the endothelium. p120 catenin plays an essential role in the regulation of cadherins, and increased endothelial adhesion mediated by this protein (Anastasiadis and Reynolds, 2000) has been proposed to regulate the actin cytoskeleton via Rho GTPases (Anastasiadis, 2007). In addition, evidence indicates that the interaction between p120 catenin and cadherins promotes AJ stability (Xiao et al., 2003; Kowalczyk and Reynolds, 2004; Oas et al., 2010). Previous studies have shown that RNAi-mediated silencing of p120 catenin results in the loss of cadherin expression and subsequent destabilization of AJs in the microvasculature (Davis et al., 2003; Xiao et al., 2003). The binding of p120 catenin to VE-cadherin prevents clathrin-dependent endocytosis, as the cadherin-catenin system remains intact in the membrane. In addition, cytoplasmic p120 catenin that is dissociated from VE-cadherin acts as a regulator of Rho GTPase activity, which is involved in cell migration and morphological changes via pseudopodia and stress fiber formation (Anastasiadis and Reynolds, 2001). A recent study has suggested the fine regulation of endothelial cell adhesion by AJs, in which p120 catenin plays a critical role in regulating the distribution of cadherins in lipid rafts of endothelial membranes, which has consequences on the stabilization of cadherins and cell signaling (Gentil-Dit-Maurin et al., 2010). The finding that the deletion of p120 catenin from skin increases inflammation via RhoA-NF-κB has important implications for diseases in humans (Perez-Moreno et al., 2006). The NF-κB pathway is involved in cell survival, inflammation, and often tumor progression (Cozzolino et al., 2003; Perez-Moreno et al., 2006). Finally, these experimental findings, together with our results (Céspedes-Rubio et al., 2010), suggest p120 catenin as a critical biomarker of the recovery of the NVU after cerebral ischemia.

## Neuron-to-astrocyte control of neurovascular coupling

Originally, astrocytes were considered supporting cells that play no role in neurotransmission in the CNS. However, studies have shown that astrocytes display a large conductance of potassium ions (K^+^) during an action potential in presynaptic neurons. K^+^ passes into the blood vessel via gap junctions for removal after a long period of neuronal activity (Massey et al., 2004; Wallraff et al., 2004; Jabs et al., 2005). Previous studies have shown that this passive role of astrocytes in the synapse changes over time, as hippocampal astrocytes in culture respond to chemical transmitters, such as glutamate (neurotransmitter), generating a Ca^2+^ wave that propagates to surrounding astrocytes (Leybaert et al., 1998; Cotrina et al., 2000). Initially, glutamate was shown as a propagator of waves in astrocytes. However, other investigations have suggested that norepinephrine, GABA, acetylcholine, histamine and adenosine induce Ca^2+^ elevation in glial cells derived from *ex vivo* preparations of adult brain slices (Duffy and Macvicar, 1995; Porter and McCarthy, 1995; Kulik et al., 1999; Shelton and McCarthy, 2000; Bowser and Khakh, 2004). Some of the signals in the neuron-to-astrocyte direction are mediated by metabotropic receptors, such as mGluR1/5. The activation of these receptors by glutamate released at synapses induces phospholipase C (PLC) activation, IP3 formation and, subsequently, IP3R activation, which facilitates the release of calcium into the cytosol from the ER (Kawabata et al., 1996; Wang et al., 2000). Calcium waves transmit signals between astrocytes, as this Ca^2+^ signal propagates to neighboring astrocytes via the diffusion or release of IP3 and the subsequent activation of purinergic P2Y receptors in neighboring astrocytes (Venance et al., 1997; Guthrie et al., 1999). Finally, the discovery of gliotransmitters (including glutamate, D-serine and ATP), chemicals released from astrocytes that affect the transmission between pre- and post-synaptic neurons, led to the concept of the tripartite synapse, which states that the activation of astrocytes associated with increased intracellular Ca^2+^ results in the direct activation of the neighboring neurons (Fiacco and Mccarthy, 2004; Gordon et al., 2005; Panatier et al., 2006). Neuronal activation by astrocytes is thought to occur via NMDA receptors that are primarily composed of NR2B subunits and is directly involved in neuronal plasticity (Fellin et al., 2004; Massey et al., 2004). Recently, studies using conditional knockout of CDK5 in the adult mouse brain showed improved performance in spatial learning tasks and enhanced hippocampal long-term potentiation via NR2B-mediated excitatory postsynaptic currents (Hawasli et al., 2007; Plattner et al., 2014). This finding, in association with the finding that gene silencing of CDK5 decreased neuropathologic characteristics of neurodegeneration, such as neurofibrillary tangles (Piedrahita et al., 2010), may suggest that the use of RNAi-mediated suppression of CDK5 expression in astrocytes promotes the tripartite synapse and recovery from cognitive impartment after stroke.

Concomitant with the function of astrocytes in the regulation of synapses, these cells directly regulate cerebral circulation, as they modulate the level of neural activity and, consequently, that of local microcirculation (Roy and Sherrington, 1890; Friedland and Iadecola, 1991; Pellerin and Magistretti, 1994). Sites of synaptic transmission that display high-energy demand during neuronal activity also display increased blood flow, which results in the rapid expansion of arterioles and capillaries (Lou et al., 1987; Magistretti et al., 1999; Kasischke et al., 2004). The propagation of calcium waves in astrocytes via glutamate receptors has been proposed to regulate blood flow during neuronal activity because these cells secrete vasoactive substances, such as NO, the product of iNOS, factors derived from the activity of cyclooxygenase (COX) and epoxygenase, such as prostaglandin E2 (PGE2), epoxyeicosatrienoic acid, and ATP, towards neurons, astrocytes, and ECs (Oomagari et al., 1991; Alkayed et al., 1997; Wiencken and Casagrande, 1999; Arcuino et al., 2002). Neuron-to-astrocyte activation via the activity of Ca^2+^-sensitive phospholipase A2 and the accumulation of arachidonic acid can induce vasodilation or vasoconstriction through its metabolic pathways. COX-2-dependent accumulation of PGE2 leads to vasodilation, whereas diffusion of arachidonic acid into smooth muscle leads to the accumulation of 20-HETE, causing vasoconstriction (Bezzi et al., 1998; Harder et al., 2002). This phenomenon reflects neurovascular coupling and has been defined as vasomotion (Filosa et al., 2004), in which the microvascular hemodynamics are regulated by the energy and oxygen demand to an undersupplied organ, i.e., low O_2_ conditions. This event triggers astrocytes to induce vasodilation in the endothelium to promote the entry of nutrients and O_2_ (Ward et al., 2000; Brown et al., 2002; Lovick et al., 2005). This bifunctional role of astrocytes in neurovascular coupling and synaptic transmission implicates these cells as important targets for functional recovery of the NVU, including neurons, glia, and ECs, after cerebral ischemia.

## Astrocytes and modulation of excitotoxicity

Astrocytes, the most abundant CNS cell population, are a type of glial cell that performs diverse functions, such as providing trophic and metabolic support to neurons (Rouach et al., 2008; Dienel, 2013), participating in differentiation, neuronal polarity, and synaptogenesis during development (new synapse formation) (Allen, 2013) and facilitating synaptic processes (Ullian et al., 2001; Allen and Barres, 2005; Kucukdereli et al., 2011). These functions render astrocytes as essential for the maintenance of brain homeostasis (Parpura and Verkhratsky, 2012) and neuronal survival (Sofroniew, 2005). In recent years, it has been determined that astrocytes participate in cerebral recovery following cell damage, particularly following damage caused by cerebrovascular diseases (CVD) or neurodegenerative disease. In such injuries, a common process that induces cell death is glutamate-mediated excitotoxicity (Matute et al., 2007; Guimarães et al., 2010). Astrocytes influence neuronal survival following brain injury via glutamate uptake, free radical removal, water transport, and cytokine and NO production. Glutamate uptake is a process in which astrocytes absorb extracellular glutamate via highly expressed glutamate transporters to maintain transmitter homeostasis (Rothstein et al., 1996; Bush et al., 1999; Swanson et al., 2004). Glutamate transporters play a major role in maintaining brain homeostasis, and the astrocytic excitatory amino acid transporters (EAATs) EAAT1 and EAAT2 are functionally dominant. Astrocytic EAATs play important roles in various neuropathologies in which astrocytes undergo cytoskeletal changes (Zagami et al., 2009; Lau et al., 2010). In addition, after brain damage, astrocytes assist in the recovery of plasticity processes, such as long-term potentiation (LTP), via the expression of trophic and molecular factors on their surface that favor neuronal growth or regeneration and new synapse formation (Matute et al., 2007; Adelson et al., 2012; Lin et al., 2014; Wang et al., 2014).

Astrocytes respond to brain injury via a process called reactive astrogliosis (Sofroniew and Vinters, 2010), in which astrocytes change their morphology and metabolism, converting from a fibrillary or protoplasmic morphology to a hypertrophic or swollen structure, which is primarily characterized by the elevated expression of intermediate filaments and proteins related to metabolic acidity. Over time, this astroglial hyperreactivity process creates a scar that impedes NVU repair; thus, reactive astrogliosis is considered an important marker of structural injury (Di Giovanni et al., 2005; Buffo et al., 2010). In an NVU homeostasis context, astrocytes are star-shaped (fibrillar) and have many extensions irradiating from the soma towards neighboring cells, which facilitate their performance of functions involving neuronal migration, support, and maintenance (Zonta et al., 2003; Sofroniew, 2005; Wang and Bordey, 2008; Buffo et al., 2010). In contrast, in a pathological context, such as ischemia, astrocytes near the injured area change, acquiring a fibrous appearance and thick extensions (Xiong et al., 2011; Barreto et al., 2012). The morphological changes in astrocytes are primarily mediated by actin cytoskeleton remodeling (Hall, 1998; Hall and Nobes, 2000). Furthermore, this actin cytoskeleton repair system is mediated by Rho GTPases (Bustelo et al., 2007). Recently, it has been demonstrated that RhoA/ROCK activity induces a protoplasmic morphology in primary astrocyte cultures, as opposed to the function of Rac1, which favors stellation or the generation of new protrusions in astrocytes (Racchetti et al., 2012). In addition, ROCK plays a major role in determining the cell surface expression of EAAT1/2, providing evidence for an association between transporter function and astrocytic glutamate uptake. ROCK inhibitors elevate glutamate transporter function; this activity profile may contribute to their beneficial effects on neuropathologies (Lau et al., 2011). In contrast, the role of Rac1 in astrocytes is associated with not only their stellar morphology but also their survival. A study conducted using human glioma cells demonstrated that suppressing Rac1 activity induces apoptosis, in contrast to the results using primary astrocytes from humans (Senger et al., 2002). This evidence suggests that Rac1 regulates certain pro-survival pathways that are abnormally activated in this type of tumor. In addition, Rac activation by PI3K has been associated with PI3K/Akt activation, which promotes cell survival (Dey et al., 2008; Linseman and Loucks, 2008; Read and Gorman, 2009). Similarly, the Rac1 activator Tiam performs an important function in astrocyte polarization and migration during development or cell repair by affecting the organization of the microfilament and microtubule networks (Ellenbroek et al., 2012). Alternatively, cellular senescence is a tumor-suppressive process that is characterized by irreversible cell cycle exit, a unique pathway that is upregulated by CDK5 activation. The increased CDK5 activity further reduces GTPase Rac1 activity and Pak activation. The repression of GTPase Rac1 activity by CDK5 that is required for the expression of the senescent phenotype may suggest CDK5-mediated Rac1 suppression as a marker of cell death (Alexander et al., 2004).

Finally, filamentous tau aggregates have been detected in astrocytes in human disease and in animal models, and this process disturbs glutamate uptake and other astrocyte functions, leading to focal neurodegeneration (Forman et al., 2005; Dabir et al., 2006). GSK3β and Cdk5 kinase activity, which regulate tau phosphorylation, are also increased in astrocytes of the senescence-accelerated mice prone (SAMP8) model. Inhibition of GSK3β using lithium or inhibition of CDK5 using roscovitine has been shown to reduce tau phosphorylation at Ser396. Moreover, a reduced mitochondrial membrane potential in SAMP8 mouse astrocytes suppresses glutamate uptake in astrocytes, which is a critical neuroprotective mechanism (García-Matas et al., 2008).

## A possible therapeutic strategy for NVU modulation

**Figure 2:**
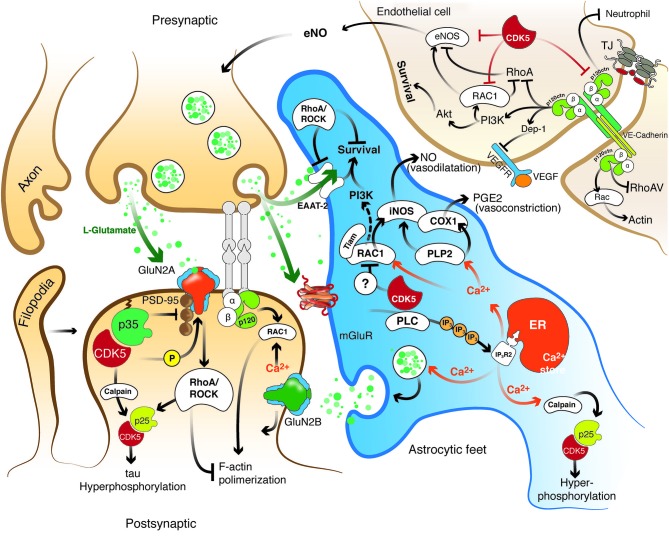
**Hypothetical model of the role of the effectors Rho GTPase, p120 catenin, and CDK5 in the recovery of the structure and function of the NVU after cerebral ischemia**. CDK5 and RhoA/ROCK targeted to ECs, astrocytes and neurons may exert protection against excitotoxicity and promote neuronal plasticity and adhesion integrity. Reduction of CDK5 expression in neurons and astrocytes may facilitate synaptic plasticity, which requires NR2B for LTP and Rac1 and p120 catenin signaling for dendritic spine morphogenesis and synaptic adhesion. Decreased expression of CDK5 induces LTP via NR2B (GluN2B)-mediated excitatory postsynaptic currents, and the consequent induction or accumulation of p120ctn inhibits RhoA/ROCK, preventing F-actin retraction and inducing Rac1 activation, thus facilitating neurotransmission. In addition, CDK5 inhibition may prevent tau hyperphosphorylation and facilitate glutamate uptake in astrocytes, preserve actin remodeling and promote angiogenesis via eNOS activation. In turn, ROCK inhibition also prevents tau hyperphosphorylation and dendritic spine retraction and increases the expression of EAATs to enhance glutamate uptake and promote BBB integrity. Moreover, actin remodeling induced by Tiam/Rac1 favors stellation and survival in astrocytes. Likewise, the signaling molecules VE-cadherin and p120 catenin activate Rac/PI3 kinase and suppress RhoA to maintain the survival and organization of the actin cytoskeleton, form complexes with VEGFR-2 to promote BBB adhesion and integrity, and prevent neutrophil diapedesis. ROCK and CDK5 targeting may promote calcium wave stimulation via glutamate receptor and PLC/IP3 signaling in astrocytes to facilitate NO and PGE2 release, thus regulating endothelial vasodilatation and vasoconstriction.

Our research group found that CDK5 gene silencing reduces histopathological markers associated with cognitive disorders (PHF-1), preventing the neurodegeneration and neuronal loss in AD mice (Piedrahita et al., 2010). We also found that Rho GTPases (Rac and RhoA) are involved in both damage and survival signaling mechanisms. For example, our recent studies show that inhibition of RhoA/ROCK using Y27632 blocks the cascade of neurodegeneration triggered by cerebral infarction, prevents the deregulation of kinases involved in neuronal cytoskeletal remodeling, such as CDK5, favors synaptic connectivity and protects against cognitive deterioration (Castro-Alvarez et al., 2011). Fasudil, Y27632, and other ROCK inhibitors have previously been proposed as potential treatments for atherosclerosis and vascular disease (Zhou et al., 2011). Complementarily, we found that Rac and synaptic adhesion proteins (p120 catenin and N-catenin) are critical for the recovery from and protection against brain infarction (Céspedes-Rubio et al., 2010; Gutiérrez-Vargas et al., 2010; Posada-Duque et al., 2013). Stroke and AD are associated with altered morphology or loss of dendritic spines (Fiala et al., 2002; Brown et al., 2008; Li and Murphy, 2008). At the postsynaptic terminal of an excitatory glutamatergic synapse in the mouse hippocampus, CDK5 phosphorylates the N-terminal domain of PSD95 to regulate the synaptic recruitment and clustering of ion channels, particularly K^+^ channels and NMDA receptors (NMDARs; Fu et al., 2001; Morabito et al., 2004; Hawasli et al., 2007). The proteins identified in the postsynaptic density include cell surface receptors, cytoplasmic signaling enzymes, and cytoskeletal and scaffold proteins. These proteins include the Rho GTPases (Jordan et al., 2004; Peng et al., 2004) RhoA, Rac1, and Cdc42, which regulate the actin cytoskeleton. Actin is the primary cytoskeletal component of dendritic spines. Accordingly, the Rho family of GTPases has been implicated in the regulation of dendritic spine morphogenesis (Govek et al., 2005; Tada and Sheng, 2006). In particular, RhoA activation exerts a strong inhibitory effect on spine morphogenesis and causes a profound loss of spines, in contrast to Rac and Cdc42 activity, which promote dendritic spine morphogenesis (Govek et al., 2005). p120ctn, a member of the cadherin/catenin system, regulates Rho GTPases to modulate actin cytoskeletal remodeling during dendritic spine formation (Togashi et al., 2002; Elia et al., 2006; Kwiatkowski et al., 2007). p120ctn also regulates spine length via Rho GTPases, whereas the control of head width requires interactions with cadherin (Elia et al., 2006; Lee et al., 2008; Ishiyama et al., 2010). In addition, the postsynaptic membrane contains a high concentration of NMDARs and associated signaling proteins, which are assembled via scaffolding proteins into the postsynaptic density. Excitotoxicity contributes to the pathogenesis of stroke, and synaptic dysfunction represents an early step in the pathology of dementia (Kamenetz et al., 2003; Walsh and Selkoe, 2004). Therefore, based on the aforementioned results and in an effort to find solutions to health issues, such as cerebral infarction, these studies suggest that Rho GTPases, p120 catenin, and CDK5 are primary effectors that function in coordination in the recovery of the structure and function of the NVU after cerebral ischemic infarction in a cell type-specific manner (Figure 2). These targets act in various neuroprotective contexts, such as AD, post-ischemic and excitotoxicity murine models. Treatment with CDK5 RNAi, roscovitine or atorvastatin improves motor, cognitive and sensory function in the post-ischemic brain and triggers neuronal plasticity and survival. The intrinsic plasticity recovery after stroke, in accordance with the results using injury models in our previous studies, may suggest that the regulation of Rho GTPases are associated with actin cytoskeletal remodeling; p120ctn and synaptic adhesion proteins expression, together with β-catenin and N-catenin, at post-synapses improve neurotransmission. Therefore, future efforts must focus on individually targeting the structural and functional roles of each effector and the interactions in neural (astrocytes, microglia, and neurons) and non-neural cells (pericytes and ECs) in the post-ischemic brain and address how to promote the recovery or prevent the loss of homeostasis in the short, medium and long term.

## Conflict of interest statement

The authors declare that the research was conducted in the absence of any commercial or financial relationships that could be construed as a potential conflict of interest.
